# Radical stereotactic radiosurgery with real-time tumor motion tracking in the treatment of small peripheral lung tumors

**DOI:** 10.1186/1748-717X-2-39

**Published:** 2007-10-22

**Authors:** Brian T Collins, Kelly Erickson, Cristina A Reichner, Sean P Collins, Gregory J Gagnon, Sonja Dieterich, Don A McRae, Ying Zhang, Shadi Yousefi, Elliot Levy, Thomas Chang, Carlos Jamis-Dow, Filip Banovac, Eric D Anderson

**Affiliations:** 1 Department of Radiation Medicine, Georgetown University Hospital, Washington, DC. USA; 2 Division of Pulmonary, Critical Care and Sleep Medicine, Georgetown University Hospital, Washington, DC. USA; 3 Biostatistics Unit, Lombardi Comprehensive Cancer Center, Georgetown University Medical Center, Washington, DC. USA; 4 Division of Vascular & Interventional Radiology, Georgetown University Hospital, Washington, DC. USA

## Abstract

**Background:**

Recent developments in radiotherapeutic technology have resulted in a new approach to treating patients with localized lung cancer. We report preliminary clinical outcomes using stereotactic radiosurgery with real-time tumor motion tracking to treat small peripheral lung tumors.

**Methods:**

Eligible patients were treated over a 24-month period and followed for a minimum of 6 months. Fiducials (3–5) were placed in or near tumors under CT-guidance. Non-isocentric treatment plans with 5-mm margins were generated. Patients received 45–60 Gy in 3 equal fractions delivered in less than 2 weeks. CT imaging and routine pulmonary function tests were completed at 3, 6, 12, 18, 24 and 30 months.

**Results:**

Twenty-four consecutive patients were treated, 15 with stage I lung cancer and 9 with single lung metastases. Pneumothorax was a complication of fiducial placement in 7 patients, requiring tube thoracostomy in 4. All patients completed radiation treatment with minimal discomfort, few acute side effects and no procedure-related mortalities. Following treatment transient chest wall discomfort, typically lasting several weeks, developed in 7 of 11 patients with lesions within 5 mm of the pleura. Grade III pneumonitis was seen in 2 patients, one with prior conventional thoracic irradiation and the other treated with concurrent Gefitinib. A small statistically significant decline in the mean % predicted DLCO was observed at 6 and 12 months. All tumors responded to treatment at 3 months and local failure was seen in only 2 single metastases. There have been no regional lymph node recurrences. At a median follow-up of 12 months, the crude survival rate is 83%, with 3 deaths due to co-morbidities and 1 secondary to metastatic disease.

**Conclusion:**

Radical stereotactic radiosurgery with real-time tumor motion tracking is a promising well-tolerated treatment option for small peripheral lung tumors.

## Introduction

Treatment options for medically inoperable patients with lung cancer are limited. Poor outcomes with protracted conventionally fractionated radiotherapy approaches prompted researchers in the last decade to explore ways of delivering high doses of radiation in shorter periods of time [1]. Utilizing a body frame and abdominal compression to limit lung motion, small mobile lesions have been treated with relatively tight margins (10 mm) [2]. This enhanced accuracy has facilitated the safe, swift delivery of highly effective doses of radiation to small discrete peripheral lung tumors such as stage I lung cancer and pulmonary metastases [3-13]. Recently updated outcomes of a Phase I stereotactic body radiotherapy (SBRT) dose escalation study confirm that abbreviated radiosurgery treatment courses, in which doses in the range of 45 Gy to 60 Gy are delivered in less than 2 weeks, result in durable local control rates ranging from 70 to 90% [14]. Such favorable outcomes establish thoracic stereotactic radiosurgery as a new radical treatment option for small peripheral lung tumors.

The CyberKnife frameless image-guided robotic radiosurgery system (Accuray Incorporated, Sunnyvale, CA) has been successfully employed at Georgetown University Hospital to treat stationary extracranial tumors since early 2002 [15]. With the introduction of the Synchrony motion tracking module, in mid 2004, tumors that move with respiration have been treated without potentially uncomfortable methods to compensate for respiratory movement, such as stereotactic body frames with abdominal compression devices and respiratory gating techniques [16]. Synchrony, an automated CyberKnife image-guidance subsystem, continuously points the robot-mounted linear accelerator at lung tumors as they move with uninhibited respiration during radiation delivery [17]. We report preliminary clinical outcomes from 24 consecutive patients with single small peripheral lung tumors radically treated using Synchrony real-time tumor motion tracking.

## Methods and materials

### Eligibility

This study was approved by the hospital institutional review board and all participants provided informed written consent. The Georgetown University Hospital multidisciplinary thoracic oncology team evaluated patients. Mandatory baseline studies included CT scans of the chest, abdomen and pelvis with IV contrast, PET imaging and routine pulmonary function tests (PFTs). Patients with small peripheral pathologically confirmed inoperable Stage I lung cancer or single pulmonary metastases were treated. Tumors were considered small if the maximum diameter measured less than 4 cm and peripheral if radical treatment was feasible without exceeding conservative maximum point dose limits to critical central normal tissues derived from historical data (Table 1). Conventional thoracic irradiation was permitted if it was delivered more than one year prior to stereotactic radiosurgery and directed to a different lobe of the lung and/or the extrapulmonary thoracic lymphatics (i.e., hilar, mediastinal and supraclavicular lymph nodes). Concurrent and salvage systemic therapies other than gemcitabine were also permitted.

**Table 1:** Critical central thoracic structure point dose limits

**Critical Structure**	**Maximum Point Dose Limit (Gy) (total for 3 fractions)**
Spinal cord	18
Left ventricle	18
Esophagus	24
Main bronchus	30
Trachea	30
Aorta	30

### Fiducial placement

Tracking based on translational and rotational target information requires that a minimum of 3 non-collinear fiducials be placed in such a way that they do not obscure each other on the orthogonal images of the CyberKnife x-ray targeting system. Therefore, 3 to 5 gold fiducials measuring 0.8–1 mm in diameter by 3–7 mm in length (Item 351-1 Best Medical International, Inc., Springfield, VA) were placed in or near the tumors under CT-guidance as recently described [18].

### Treatment planning

Fine-cut (1.25 mm) treatment planning CTs were obtained 7–10 days after fiducial placement during a full inhalation breath hold with the patient in the supine treatment position. This short delay prior to imaging allowed procedure-related hemorrhage to resolve and limited post-CT fiducial migration. Gross tumor volumes (GTV) were contoured utilizing lung windows. All critical central thoracic structures (Table 1) and the lungs were contoured. A treatment plan with a 5-mm margin on the GTV for contouring and tracking uncertainty was generated using the TPS 5.2.1 non-isocentric, inverse-planning algorithm with tissue density heterogeneity corrections for lung based on an effective depth correction. Radical doses of 45 to 60 Gy in three equal fractions of 15 to 20 Gy were prescribed to an isodose line that covered at least 95% of the planning treatment volume (PTV = GTV + 5 mm). In general, total doses closer to 45 Gy were prescribed when concerns about the radiation tolerance of adjacent critical structures arose and when patients were felt to have severe cardiopulmonary dysfunction. The percentage of the total lung volume receiving 15 Gy or more (V15) was limited to less than 15% in order to decrease the risk of clinically significant radiation pneumonitis or pulmonary dysfunction.

### Treatment delivery

The treatment course was completed in less than two weeks. Prior to the initial treatment, each patient was evaluated with fluoroscopy to verify that the motion of the fiducials chosen for tracking correlated with tumor motion. Prophylactic corticosteroids were not administered. Patients were placed supine and unrestrained on the treatment table with their arms at their sides. They wore a form-fitting vest upon which 3 red light-emitting markers were attached on the surface of the patient's anterior torso in the region of maximum respiratory excursion of the chest and upper abdomen. These markers projected to an adjustable camera array in the treatment room. Precise patient positioning was accomplished utilizing the automated patient positioning system. Fiducials were located using orthogonal x-ray images acquired with ceiling-mounted diagnostic x-ray sources and corresponding amorphous silicon image detectors secured to the floor on either side of the patient.

Immediately prior to treatment delivery, an adaptive correlation model was created between the fiducial positions as periodically imaged by the x-ray targeting system and the light-emitting markers as continuously imaged by the camera array [17]. During treatment delivery the tumor position was tracked using the live camera array signal and correlation model, the linear accelerator was moved by the robotic arm in real time to maintain alignment with the tumor during uninhibited respiration. Fiducials were imaged prior to the delivery of every third beam for treatment verification and to update the correlation model [16]. If fiducials were misidentified by the software or the correlation model error exceeded 3 mm in two consecutive paired x-ray images, treatment was discontinued and the correlation model rebuilt.

### Follow-up studies

Patients were followed with physical examinations, CT imaging and routine PFT's at 3, 6, 12, 18, 24 and 30 months. Complete response was defined as resolution of the tumor on CT imaging and partial response as a decrease in the tumor volume relative to the treatment planning CT. Local and regional tumor recurrence was defined as unequivocal tumor progression on CT imaging within the treated lobe or regional lymph nodes, respectively. Biopsy was recommended for pathologic verification. Toxicity was scored according to the National Cancer Institute Common Terminology Criteria for Adverse Events, Version 3.0 [19].

### Statistical analysis

Follow-up was determined from the date of the last treatment. Two-sided Wilcoxon signed-ranks tests were used to assess statistical significance (α = 0.05) of post-treatment changes in forced expiratory volume in 1 sec (FEV1), total lung capacity (TLC) and diffusing capacity of the lung for carbon monoxide (DLCO) at 6 and 12 months.

## Results

### Patient and tumor characteristics

Twenty-four consecutive patients (10 men and 14 women) were treated over a 2-year period extending from July 2004 to July 2006 (Table 2). The median follow-up time among survivors is 12 months (range, 6–30 months). No patients were lost to follow-up. Seventeen percent of patients received prior conventional thoracic radiation. All but one patient had stopped smoking in the distant past (> 3 years) or had never smoked. Nonetheless, pulmonary dysfunction was the primary rationale for non-surgical treatment among the stage I lung cancer patients and 3 such patients required supplemental home oxygen prior to receiving treatment. Sixty-seven percent of the tumors involved the upper lobes. Fifteen were inoperable primary lung tumors (adenocarcinoma 7, NSCLC not otherwise specified 5, squamous cell carcinoma 2 and typical carcinoid tumor 1) and 9 were single lung metastases (NSCLC 5, esophagus 1, small bowel 1, renal 1 and cutaneous basal cell carcinoma 1). The mean maximum tumor diameter was 2 cm (range, 0.9 – 3.5 cm).

**Table 2:** Patient and Tumor Characteristics

	**Mean (Range)**
Age (years)	70 (50 – 82)
Weight (lbs)	160 (118 – 285)
FEV1 (L)	1.47 (0.53 – 2.62)
% predicted FEV1	61 (26 – 121)
% predicted TLC	94 (69 – 136)
% predicted DLCO	61 (44 – 96)
Maximum Tumor Diameter (cm)	2.0 (0.9 – 3.5)
Gross Tumor Volume (cc)	8 (1 – 14)

### Treatment characteristics

Three equal fractions of 15 to 20 Gy were delivered in an average of 7 days (Table 3). Treatment plans were composed of hundreds of pencil beams shaped using a single 20, 25 or 30-mm diameter circular collimator. The percentage of the total lung volume receiving 15 Gy or more was low despite the radical treatment intent. On average, 55 paired x-ray images were taken each day to confirm the accuracy of the correlation model. Twenty-five percent of the patients received concurrent systemic therapy as previously described [20].

**Table 3:** Treatment Characteristics

	**Mean (Range)**
Dose (Gy)	54 (45 – 60)
Biologic Effective Tumor Dose (BED Gy_10_)	150 (110 – 180)
Prescription Isodose Line (%)	80 (75 – 90)
Planning treatment volume coverage (%)	97 (95 – 100)
Number of beams per fraction	164 (87 – 270)
Number of paired x-ray verification images per fraction	55 (29 – 90)
Beam-on time (minutes)	82 (53 – 120)
Treatment course (days)	7 (3 – 11)
% Total lung volume receiving 15 Gy or more	7 (3 – 11)

### Complications

Pneumothorax either during or immediately following fiducial placement was seen in 30% of patients, and 17% of all patients required tube thoracostomy to correct clinically significant pneumothorax. All patients completed treatment without interruption. Following treatment, acute toxicity consisting of mild brief fatigue was reported in the majority of patients. Transient chest wall discomfort, typically lasting several weeks, developed in 7 of 11 patients with lesions within 5 mm of the pleura.

Grade III pneumonitis was observed in 2 patients (8%). One of the patients received concurrent Gefitinib treatment. She developed an infiltrate corresponding to the high dose stereotactic radiosurgery volume and dyspnea requiring temporary supplemental oxygen 4 weeks after completing CyberKnife treatment. Her symptoms resolved quickly with steroids and the discontinuation of Gefitinib. The second patient, who had a history of extensive conventional esophageal irradiation, was treated for a single lung metastasis. He developed symptomatic infiltrates largely confined to the conventional radiation volume following the initiation of salvage experimental systemic therapy 10 months after radiosurgery. His symptoms resolved over several weeks on steroids and he discontinued supplemental oxygen.

### Post-treatment pulmonary status

Among the entire group, no change was seen in FEV1 and TLC at 6 and 12 months. A statistically significant decline of 8% (from 61% to 53%; p = 0.002) and 10% (from 61% to 51%; p = 0.01) in the mean % predicted DLCO was seen at 6 and 12 months, respectively.

### Tumor response

**Figure 1:**
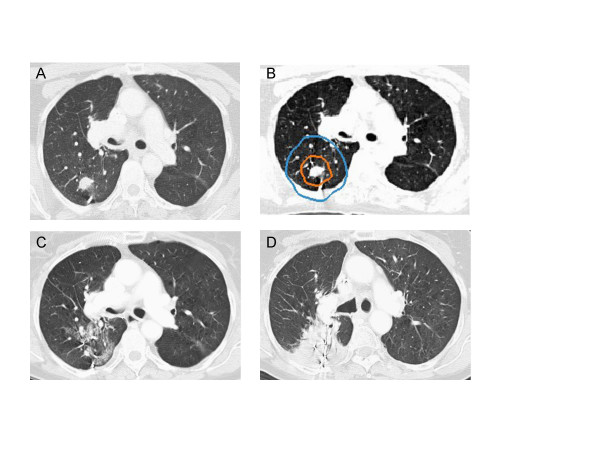
Right upper lobe clinical stage IA NSCLC treatment planning CT (A), planned radiation dose distribution (B: the planning treatment volume is shown in orange and the 30 Gy isodose line in blue), and CT at 6 and 12 months post-treatment (C and D) show progressive fibrosis in the treated volume that ultimately impedes CT evaluation of tumor response.

All tumor volumes were reduced on CT imaging at 3 months. Six-month CT scans were available for all 24 patients. Fourteen lesions continued to respond to treatment, three of which had resolved completely. Ten lesions were obscured by radiation fibrosis at 6 months and were not clearly evaluable. At 12 months, 16 patients' CT scans were available for review. Four of the evaluable lesions had responded completely, two exhibited an excellent partial response to treatment and eight, or 50% of the evaluable lesions, were obscured by radiation fibrosis which corresponded with the planned high-dose treatment volume and consistently encompassed the fiducials (Figure 1). Despite the development of significant radiation fibrosis with time, it was clear that two single lung metastases had progressed locally per CT imaging at 12 months (Table 4). Therefore, with a median follow-up of 12 months, the crude local control rate for the group is 92%. Consistent with other reports, local control was 100% for stage I tumors and lower (78%) for single lung metastases (Table 5) [21].

**Table 4:** Tumor response per CT imaging

	**6 months (%)**	**12 months (%)**
Complete Response	12	25
Partial Response	46	13
Obscured by Fibrosis*	42	50
Local Progression	0	12

* no evidence of progression

**Table 5:** Crude Local Control and Survival Rates at a Median Follow-up of 12 months

	Crude Local Control Rate (%)	Crude Survival Rate (%)
Stage I Lung Cancer	100	87
Single Lung Metastases	78	78
Overall	92	83

### Disease spread and survival

Regional lymph node failure was not observed in early follow-up. Four patients with locally controlled single lung metastases developed additional metastatic sites and received salvage systemic therapy. Despite treatment one patient died of progressive metastatic disease at 8 months. A second single lung metastasis patient died of a myocardial infarction at 11 months without evidence of local or systemic disease. No stage I lung cancer patient developed metastatic disease. However, 2 stage I lung cancer patients died of comorbid illnesses (1 secondary to progressive congestive heart failure at 6 months and 1 secondary to progressive emphysema at 9 months). Therefore, with a median follow-up of 12 months, the crude survival rate for the group is 83%, with 3 deaths due to co-morbidities and 1 secondary to metastatic disease. As expected, the crude survival rate for patients with single lung metastases was lower (Table 5) [21].

## Discussion

In mid-2004 we initiated a frameless image-guided high-dose fractionated stereotactic radiosurgery treatment protocol for patients with medically inoperable small peripheral stage I lung cancer and single small peripheral lung metastases. Continuous tracking of respiratory tumor motion with Synchrony and highly accurate beam alignment throughout treatment with the CyberKnife prompted us to deliver dose distributions with tighter margins than historically feasible (5 mm) [2]. Hundreds of beams were used to produce a relatively high central tumor dose and dose gradients that conformed closely to the shape of the tumors [22]. Twenty-four patients have been treated in 24 months without notable discomfort during the treatment procedure. With a median follow-up of 12 months the crude local control rate is 92% and there have been no severe (grade IV) treatment-related complications or mortalities. Thus, we conclude that radical stereotactic radiosurgery with real-time tumor motion tracking and continuous beam correction utilizing the CyberKnife system is a feasible, well-tolerated and highly effective treatment option for small peripheral lung tumors.

Despite promising early results, critical issues concerning the evaluation of treatment efficacy and the possibility of late complications have yet to be fully addressed. High-dose radiation delivered precisely to small peripheral pulmonary nodules will cause focal lung parenchyma fibrosis that complicates interpretation of tumor response. At 3 months all tumors had responded to treatment, as seen by a decrease in volume on CT imaging. However, at 12 months half of the lesions were obscured by radiation fibrosis conforming to the high-dose radiation volume, making further CT tumor response assessment difficult [23,24]. In our experience, PET activity within irradiated regions does not reliably indicate tumor recurrence because the radiation response in the lung is itself PET avid. Therefore, PET imaging was not routinely used to follow patients in this study. Although biopsy could aid response assessment, it was not recommended in these typically frail patients in the absence of frank CT tumor progression given the limited salvage treatment options available. Consequently, when treated tumors appeared to be obscured by radiation-induced fibrosis on serial CT images (Figure 1), the tumors were considered locally controlled and patients were observed with the understanding that the documentation of local recurrence might be delayed.

High-dose thoracic radiotherapy delivered to small pulmonary nodules, no matter how accurate, results in limited peritumoral lung damage and dysfunction. In the absence of validated radiation pneumonitis risk parameters for stereotactic radiosurgery, we chose to simply limit the volume of lung receiving 15 Gy or greater. Although we were able to limit this volume (V15 ranged from 3% to 11% of total lung volume), Grade III pneumonitis occurred in two patients, one at 4 weeks post-treatment and the other at 10 months post-treatment. In both cases pneumonitis onset was correlated with systemic therapy, and one patient had had prior extensive conventional thoracic irradiation. Both patients recovered with steroid treatment. No patients died of pneumonitis, lung fibrosis or local recurrence; deaths in this trial were due to comorbid illness or preexisting metastatic disease progression.

Limited data are available evaluating the impact of stereotactic radiosurgery on pulmonary function in patients with small peripheral lung tumors (< 4 cm). Furthermore, available findings are difficult to interpret because a large fraction of lung cancer patients stop smoking just prior to treatment; any deleterious effects of radiosurgery may be offset by the early beneficial effects of smoking cessation [25]. Ninety-five percent of the patients in the current trial discontinued smoking in the distant past (>3 years prior to treatment) or had never smoked. The mean percentage of the total lung volume receiving a minimum of 15 Gy was 7%. As might have been anticipated given the relatively small volumes of peripheral lung irradiated to doses capable of causing local lung dysfunction, small but statistically significant 8% and 10% declines in the mean % predicted DLCO were seen at 6 and 12 months, respectively [26]. Regardless of the decline, no adverse clinical effect was observed. Furthermore, the negative impact of radiosurgery on diffusion capacity may be overestimated in the current study as this effect is expected to be greater in patients treated with prior conventional thoracic irradiation or concurrent systemic therapy [27].

Critical central structure toxicity was not observed in this trial. It is likely that toxicity was absent because we strictly adhered to conservative maximum point dose limits for critical central structures (Table 1). However, transient mild-to-moderate chest wall pain typically lasting several weeks was seen following treatment in the majority of patients with lesions within 5 mm of the pleura. These patients were treated conservatively with non-steroidal anti-inflammatory medications or opioid analgesic combinations. Although it is tempting to limit the dose delivered to the chest wall in these patients, this would likely result in additional local failures and is not recommended at this time.

The current CyberKnife treatment approach requires the implantation of fiducials to permit tumor targeting and tracking. Fiducial placement results in a delay in therapy while awaiting the resolution of procedure-related hemorrhage and fiducial fixation. Furthermore, the procedure may result in pneumothorax, sometimes requiring tube thoracostomy and a brief hospital stay [28]. Our institution has developed a technique for placing fiducials in or near central and larger peripheral tumors via bronchoscopy reducing the risk of pneumothorax [29]. However, for the small peripheral tumors treated in this study sophisticated navigation systems would be required to place fiducials precisely in this manner. Fortunately, ongoing research evaluating fiducial-less tracking will likely result in technology that obviates the need for peripheral fiducial placement in the near future [30].

## Conclusion

Small peripheral lung tumors may be radically treated with the CyberKnife frameless image-guided robotic radiosurgery system, resulting in encouraging early local control rates (92%) and minimal toxicity. The delivery of hundreds of beams while continuously tracking respiratory tumor movement and adjusting beam directions allows for highly conformal dose distributions with tight margins (5 mm). It is likely that such treatment will result in superior long term tumor control with acceptable toxicity and overall better treatment outcomes.

## Abbreviations

BED Gy_10_: biologic effective tumor dose; CT: computed tomography; DLCO: diffusing capacity of the lung for carbon monoxide; FEV1: forced expiratory volume in 1 sec; GTV: gross tumor volume; Gy: Gray; NSCLC: non-small cell lung cancer; PET: positron emission tomography; PFT: pulmonary function tests; PTV: planning treatment volume; TLC: total lung capacity; V15: total lung volume receiving 15 Gy or more.
